# Efficacy of a one-shot marbofloxacin treatment on acute pleuropneumonia after experimental aerosol inoculation of nursery pigs

**DOI:** 10.1186/s40813-018-0089-2

**Published:** 2018-06-22

**Authors:** Doris Hoeltig, Judith Rohde, Birgit Brunner, Klaus Hellmann, Erik Grandemange, Karl-Heinz Waldmann

**Affiliations:** 1 Clinic for Swine, Small Ruminants, forensic Medicine and Ambulatory Service, University of Veterinary Medicine Hannover, Foundation, Bischofsholer Damm 15, D-30173 Hannover, Germany; 2 Institute for Microbiology, University of Veterinary Medicine Hannover, Foundation, Bischofsholer Damm 15, D-30173 Hannover, Germany; 3 Klifovet AG, Geyerspergerstr. 27, D-80689 Munich, Germany; 4 Vetoquinol SA, Research and Development Centre, B.P. 189, Cedex 70204 Lure, France

**Keywords:** Enrofloxacin, Marbofloxacin, Respiratory disease, Swine, Bacteriological cure, Fluoroquinolone, Concentration-dependent activity, Pig, *Actinobacillus pleuropneumoniae*

## Abstract

**Background:**

Porcine pleuropneumonia, caused by *Actinobacillus pleuropneumoniae,* is a bacterial respiratory disease of swine. Acute outbreaks of the disease are often accompanied by high mortality and economic losses. As severe cases of the disease frequently require parenteral antibiotic treatment of the animals, the efficacy of a single, high dose of marbofloxacin was compared to a three-time application of a dose of enrofloxacin under experimental conditions.

**Methods:**

A blinded, controlled, randomized and blocked dose confirmation study was conducted to test the efficacy and safety of a single dose of 8 mg/kg marbofloxacin (160 mg/ml, Forcyl® Swine, Vetoquinol SA, France) to treat acute porcine pleuropneumonia after experimental aerosol inoculation of pigs with *A. pleuropneumoniae* serotype 2. The results were compared to a three consecutive day treatment of 2.5 mg/kg enrofloxacin and a mock (saline) treatment. Criteria for the assessment of efficacy were severity of lung lesions, bacteriological cure and the course of clinical disease after treatment.

**Results:**

Thirty six nursery pigs were divided into three treatment groups: marbofloxacin (T1), enrofloxacin (T2) and mock (T3). Statistically significant superiority (*p* < 0.05) of marbofloxacin and enrofloxacin compared to the mock-treated group was demonstrated for all efficacy criteria. The need of rescue euthanasia due to severity of symptoms was significantly reduced in both treatment groups (T1: 1 pig; T2: 0 pigs; vs. T3: 8 pigs). On day 6 after treatment initiation, clinical cure was observed in 10 (T1), 10 (T2) but only 1 of the piglets in T3. Extent of lung lesions (mean of lung lesion score T1: 3.9, T2: 6.0, T3: 21.1) and bacteriological isolation from lung tissue (on day 6 after treatment initiation: T1 = 0 pigs; T2 = 1 pig; T3 = all pigs) were also significantly reduced within both treatment groups. There were no adverse events linked to the drug administration and no injection site reactions were observed.

**Conclusions:**

Both applied antimicrobial treatments were proven safe and efficacious for the treatment of acute porcine pleuropneumonia. No statistically significant differences were detected between the antibiotic treatments.

## Background

Porcine pleuropneumonia is a respiratory disease caused by the gram-negative bacterium *Actinobacillus* (*A*.) *pleuropneumoniae.* This germ is distributed worldwide and is considered obligate pathogenic and can therefore cause severe respiratory disease without additional co-infections [1–3]. The severity of disease depends on several factors such as involved serotype, infection dose, co-infections, immune status and genetic background of the animal and other environmental factors [4–6]. The disease occurs predominantly in pigs under six months of age but pigs of all ages can be affected [7, 8]. In the last few years, an increase in clinical cases, especially in nursery pigs and replacement sows has been observed throughout Germany and in other European countries [9]. Acute outbreaks of the disease have a major impact on animal welfare as well as on profitability of the pig farms. Carcass trimming and condemnation as well as costs due to animal losses in cases of high mortality, treatment, reduced daily weight gain and a prolonged fattening period lead to high direct as well as indirect economic losses [10–12].

Currently control and prevention of the disease are mainly achieved with the administration of antimicrobials and by vaccination. However, vaccination efficacy is often hampered by limited cross-serovar protection. Furthermore, it does not prevent the colonization of the lungs. This means that pigs may still carry the pathogen and remain an important source of contagion for the spreading of the infection [13–17]. The antimicrobial treatment of pigs also has some disadvantages. One disadvantage is that despite an antibiotic treatment *A. pleuropneumoniae* might not be completely cleared from the lungs of colonized animals as was demonstrated for tulathromycin treatment [18]. Another disadvantage of antimicrobial treatments is the risk of resistance development towards antibiotic substances. Highest rates of resistance of *A. pleuropneumoniae* were detected against tetracyclines followed by sulfonamides, ampicillin and trimethoprim, whereas the lowest levels of resistance were seen against fluoroquinolones, cephalosporins and florfenicol [19–24]. Fluoroquinolones and 3rd generation cephalosporins are classified as critically important antimicrobials in human medicine [25]. Therefore their use should be limited to an inevitable minimum, administered only to diseased animals that are expected to respond poorly to other classes of antibiotics based on susceptibility testing results [26–29]. One approach for the reduction of antibiotic use is the treatment of the individual diseased animal in contrast to the treatment of the whole group. This approach is controversial, as all animals of the group might be at risk of developing the disease. Nevertheless, the individual, parenteral treatment is often necessary, especially during acute outbreaks of porcine pleuropneumonia where suffering animals may be too weak for sufficient water or feed intake leading to inadequate intake of antibiotics that are administered in the feed or drinking water. Many antibiotic products including fluoroquinolones, which are registered for the parenteral treatment of porcine pleuropneumonia, require an administration of at least three or more consecutive days [8, 30]. Fluoroquinolones have a concentration-dependent mode of action and it has been shown that a high dose given as a single injection has good efficacy against *A. pleuropneumoniae* infection [31–33]. The aim of this study was to investigate the efficacy of a one-shot 8 mg/kg marbofloxacin treatment on the development of clinical signs, lung lesions and colonisation of the lungs of piglets inoculated with *A. pleuropneumoniae,* in comparison to a mock and to a standard 3-day enrofloxacin treatment protocol to obtain marketing authorization approval for a product containing marbofloxacin.

## Methods

### Study design

The study was a blinded, controlled, randomized and blocked dose confirmation study to test the efficacy of a single dose of 8 mg/kg marbofloxacin (160 mg/ml, Forcyl® Swine, Vetoquinol SA, France) as treatment for acute porcine pleuropneumonia after experimental aerosol inoculation of piglets. The experimental and treatment unit was the individual animal.

### Animals and animal housing

A total of 36 nursery pigs, aged eight weeks were included in this study. All pigs were German hybrid pigs, male castrates, vaccinated against *M. hyopneumoniae* and PCV-2. All piglets originated from the same *A. pleuropneumoniae* free piglet producer farm and had been transferred to the experimental farm at the age of four weeks. The pigs were kept and cared for according to the principles for Protection of Vertebrate Animals used for Experimental and other Scientific Purposes European Treaty Series, nos. 123 and 170 (http://conventions.coe.int/treaty/EN/treaties/html/123.htm; http://conventions.coe.int/treaty/EN/treaties/html/170.htm). The study design and housing conditions were approved by the local governmental ethics committee (Commission for ethical estimation of animal research studies of the Lower Saxonian State Office for Consumer Protection and Food Safety; approval number: 33.9–42,502-05-14A447). The pigs were kept under standardized level 2 conditions with 8m^2^ floor space per 12 pigs and fed a standardized commercial diet.

The piglets arrived at the research unit 28 days prior to inoculation, to ensure that they were thoroughly acclimatized to the new environment, diet and clinical examination procedure. After arrival blood samples for serological testing were drawn and a physical examination was performed. From the day of arrival until day of inoculation, general health status observations of the pigs were conducted twice a day. On the day prior to inoculation, all pigs were weighed and examined. All animals entering the study were tested serologically negative for *A. pleuropneumoniae* and considered to be clinically healthy. Serological screening of the pigs was conducted using ApxIV-ELISA (IDEXX APP-ApxIV Ab Test®, Co. IDEXX Laboratories, Maine, USA).

### Experimental inoculation

The experimental inoculation was performed via aerosol following the procedure described by Jacobsen et al. [6]. Briefly, the pigs were driven calmly into an aerosol chamber in groups of six animals. The animals were nebulized with 13 ml of a suspension of *A. pleuropneumoniae* serotype 2 strain C3656 containing 5,2 × 10^7^ colony forming units (cfu). The total time of exposure was 30 min. The Minimal Inhibitory Concentrations (MIC) of marbofloxacin and enrofloxacin were determined prior to infection. For both antibiotics the MIC was 0.125 μg/ml. Thus the challenge strain was considered susceptible to fluoroquinolone antibiotics according to the CLSI clinical breakpoint of ≤0.25 μg/ml for susceptibility in *A. pleuropneumoniae* from respiratory samples from pigs [34].

### Assignment to treatment groups and inclusion criteria

Only animals that fulfilled all inclusion criteria were enrolled for treatment. Inclusion criteria were pyrexia with rectal temperature > 40.3 °C and a respiratory score ≥ 2 and a depression score ≥ 1 after the experimental inoculation. Table 1 presents the description of scoring schemes. Each individual pig that fulfilled the inclusion criteria was immediately randomized and treated directly. The randomization allocation is shown in Table 2. Randomized blocking, arranging the experimental units in groups (blocks) that were equal, was used to reduce the experimental error. Blocking size was 3 at the ratio of 1:1:1, blocking factor was sex of the pigs.

**Table 1:** Scoring schemes for the assessment of clinical signs

Scoring Points	Respiratory Score	Depression score
0	No clinical signs of respiratory disease	Active, alert, normal feed intake
1	Breathing frequency of 35–45/min and / or occasional coughing	Calm, alert, reduced feed intake
2	Breathing frequency of 46–70/min and/ or multiple coughing periods within 10 min and dyspnea	Dull, increased recumbence, increased reaction time, still moving to the feeding trough but without or only minimal feed intake or dull, sitting like a dog, increased reaction time, still moving to the feeding trough but no or only minimal feed intake
3	Breathing frequency > 70/min and cyanosis or gasping or open-mouth breathing or breathing frequency > 70/min and cyanosis and gasping or open-mouth breathing	Apathetic, no reaction to stimulation and/or shaky movements without lying down and / or standing with head down without lying down and/or vomiting and / or foam around nostrils and mouth

**Table 2:** Assignment to treatment groups and treatment procedures

Treatment Group	Active Ingredients	Application Route	Dosage [unit/kg]	Duration of treatment^a^	Number of animals^b^
T1	Marbofloxacin	IM	8 mg/kg	Day 0^c^	11
T2	Enrofloxacin	IM	2.5 mg/kg	Days 0, 1, 2	12
T3	Saline 0.9%	IM	1 ml/20 kg	Days 0, 1, 2	11

^a^after fulfilling the inclusion criteria for treatment

^b^Intention to treat populations

^c^animals of this group received 0.9% NaCl solution (1 ml/20 kg) on days 1 and 2 after first treatment

Treatments (antibiotics and/or mock) were administered on Day 0, Day 1 and Day 2 (Table 2). Enrofloxacin was chosen as a reference product for the positive control with a dose of 2.5 mg/kg/day, administered on three consecutive days. Animals in the mock treatment group (T3) received administrations of saline per kg bodyweight at the same volume as the animals treated with marbofloxacin (T1) at the same time interval. For blinding purposes, all clinical examinations and the drug administrations were carried out by different members of staff. This ensured that the person responsible for the evaluation of the clinical symptoms, and therefore efficacy of the treatment, was not aware of the treatment group the pigs were assigned to.

### Clinical examination

Starting four hours after inoculation and thereafter every two hours over the following 24 h period, the pigs were clinically examined for signs of respiratory disease until they fulfilled the inclusion criteria and received the first dose of treatment. After this first treatment administration, the pigs were examined 4, 8, 12 and 24 h ± one hour. Thereafter the clinical signs were recorded twice a day until day 7 post inoculation. The clinical examination of pigs consisted of the assessment of general appearance (including posture, behavior, feed intake, rectal temperature, presence of vomiting) and clinical signs of respiratory disease (breathing type, respiratory frequency, coughing). Results of the examination were transformed into a respiratory and a depression score (Table 1) on a scale from 0 to 3. Clinical cure was defined as a rectal temperature < 40.0 °C and absence of clinical signs of respiratory disease and no depression on study day 6 (D6). Using the body weight taken prior to inoculation and on the day of removal, the average daily weight gain of the pigs was calculated.

Additional criteria for euthanasia were determined to reduce the level of stress and suffering of the pigs. Criteria for euthanasia were multifold (Table 3). Animals that were removed prior to D6 due to severity of disease were counted as not cured.

**Table 3:** Criteria for euthanasia of animals prior to end of study

Code	Description of criteria
E1	Respiratory score of 3
E2	Depression score of 3
E3	Rectal body temperature > 42.0 °C
E4	Rectal body temperature < 37.5 °C and respiratory score > 1
E5	Rectal body temperature < 37.5 °C and depression score > 1
E6	Rectal body temperature > 40.3 °C and respiratory score > 1 on more than 2 consecutive days after day 3 post inoculation
E7	Rectal body temperature > 40.3 °C and depression score > 1 on more than 2 consecutive days after day 3 post inoculation
E8	Any unpredictable event, reaction to treatment or disease leading to a moderate to severe reduction of general condition for more than 48 h
E9	Any unpredictable event, reaction to treatment or disease inducing pain for more than 48 h

On day 7 post inoculation, or earlier in cases of withdrawal on humane grounds, the pigs were euthanized by lethal intravenous injection of 80 mg/kg pentobarbital (Euthadorm® 500 mg/ml; Co. CP Pharma GmbH, Burgdorf, Germany). Necropsy was performed directly after the death of each animal.

### Bacteriological lung examination

For the bacteriological examination, 7 lung tissue samples (approximately 1cm^2^) collected from defined areas, located in the outer third of each of the seven lung lobes (one from each lobe), were collected and examined for the presence of *A. pleuropneumoniae*. Samples were plated on Columbia sheep blood agar, chocolate agar supplemented with 0.001% NAD and *A. pleuropneumoniae*-selective blood agar [35] using the quadrant streaking method. Abundance of growth was assessed semi-quantitatively. Bacterial isolates were identified as *A. pleuropneumoniae* by amplification of the apxIV gene [36].

### Necropsy

During necropsy the macroscopic extent of the developed lung lesions was assessed. For an objective assessment the lung lesion score (LLS) specified by the European Pharmacopoeia (3rd edn. EDQM, Council of Europe, Strasbourg, France) for the testing of *A. pleuropneumoniae* vaccines [37] was used. The score is based on the recording of lung lesions after palpation and macroscopic evaluation of the lung on a schematic map of the lungs. On this map the lung is split into equal sized triangles. According to the size of the lesions a number of triangles is marked. The maximum score of each lung lobe is five, leading to a total maximum score of 35.

### Statistical analysis

All collected data were entered into a database, based upon MS Access® 2010 (Microsoft Corporation, Dublin, Ireland). Verification was assured by double data entry. All statistical operations were carried out using SAS® statistical analysis software version 9.3 (SAS Institute Inc., Cary, NC, USA). Primary criterion for efficacy testing was the assessment of the developed lung lesion. Secondary criteria for the analysis were bacteriological cure, clinical cure on day D6, evolution of clinical scores, rectal temperature, withdrawals related to respiratory disease after inoculation and daily weight gain. The safety of the treatments was analyzed based upon percentage of adverse events and percentage of injection site reactions. For all continuous variables sample size, mean (m), standard deviation (SD), median, quartiles, minimum and maximum were calculated. Categorical or binary variables were displayed as absolute and relative frequencies. For the analyses ANOVA, Fisher’s exact test and Mantel-Haenszel chi- square statistics were used. The data of the LLS were log-transformed as the non-transformed data were expected to be not normally distributed. The applied level of significance was 5% (*p* < 0.05).

## Results

An overview of the main clinical data characteristics and results is given in Table 4.

**Table 4:** Overview of results of comparative treatment analysis including main data characteristics

	Treatment Group T1	Treatment Group T2	Treatment Group T3	*p*-Values for Differences
Active component	Marbofloxacin	Enrofloxacin	0.9% saline	
Number of animals	12	12	12	
Average body weight of the animals	12.0 ± 2.4	12.6 ± 2.9	12.1 ± 2.4	
Intention to treat population (number of animals)	11	12	11	
Number of animals included for primary efficacy criterion analysis	10	12	10	
Number of animals included for secondary efficacy criteria analyses	11	12	11	
Number of removals due to euthanasia criteria (mortality %)	1 (8.3%)	0 (0.0%)	8 (66.7%)	T1:T2 = > 0.05
				T2:T3 = < 0.001
				T1:T3 = 0.008
Lung Lesion Score^a^ mean Min-Max	3.9 ± 4.1 0.0–14.9	6.0 ± 5.1 0.5–20.1	21.1 ± 7.7 10.2–35.0	T1:T2 = 0.34 T2:T3 = < 0.0001 T1:T3 = < 0.0001
Number of animals bacteriologically cured^b^ (%)	11 (100%)	1 (91.7%)	0 (0%)	T1:T2 = > 0.05
				T2:T3 = < 0.001
				T1:T3 = < 0.001
Numbers of animals clinically cured^b^ (%)	10 (90.9%)	10 (83.3%)	1 (9.1%)	T1:T2 = > 0.05
				T2:T3 = < 0.001
				T1:T3 = < 0.001
Daily weight gain^b^: infection to removal (kg)	1.81 ± 0.774	1.83 ± 0.606	0.69 ± 1.335	T1:T2 = < 0.05
				T2:T3 = < 0.05
				T1:T3 = < 0.05

^a^Primary efficacy criterion

^b^Secondary efficacy criterion

### Clinical data and inclusion for treatment

Prior to inoculation all pigs had a respiratory score and a depression score of 0 and a body temperature ≤ 40.0 °C. The last clinical examination prior to inoculation was performed one hour before the start of the experimental inoculation. Of the 36 nursery pigs included in this study, 35 animals developed typical clinical signs of porcine pleuropneumonia after challenge. One pig stayed clinically healthy without any signs of disease and another one met the criteria for euthanasia prior to treatment. These two pigs were therefore not treated, leaving 34 pigs for the intention to treat population (ITT); 11 pigs in group T1, 12 pigs in group T2 and 11 pigs in group T3. Two pigs were treated despite not fulfilling all inclusion criteria (respiratory scores of 1 instead of 2). Therefore they were excluded from the final primary efficacy criterion analyses. These pigs belonged to treatment groups T1 and T3.

The mean body weight of the ITT population prior to inoculation was 12.1 kg with an SD of 2.5 (T1: 12.0 ± 2.4; T2: 12.6 ± 2.9; T3: 12.1 ± 2.4). The median time between challenge and treatment was 5.9 h for T1, 6.6 for T2 and 5.9 for T3 with no statistical significant differences between the three treatment groups. All pigs of the ITT population had a rectal temperature ≥ 40.3 °C prior to treatment (no statistical significant differences between treatment groups). The depression score was 1 for 79.4% (27 pigs) of all included animals and 2 for 20.6% of the pigs (T1:1 pig, T2: 2 pigs and T3: 4 pigs; no statistical significant differences between the treatment groups). Seven pigs in group T3 and one in group T1 met the criteria for euthanasia and were euthanized prior to day D6. One pig in group T3 died due to the severity of the infection. An overview of the most important clinical data is shown in Table 4.

**Fig. 1:**
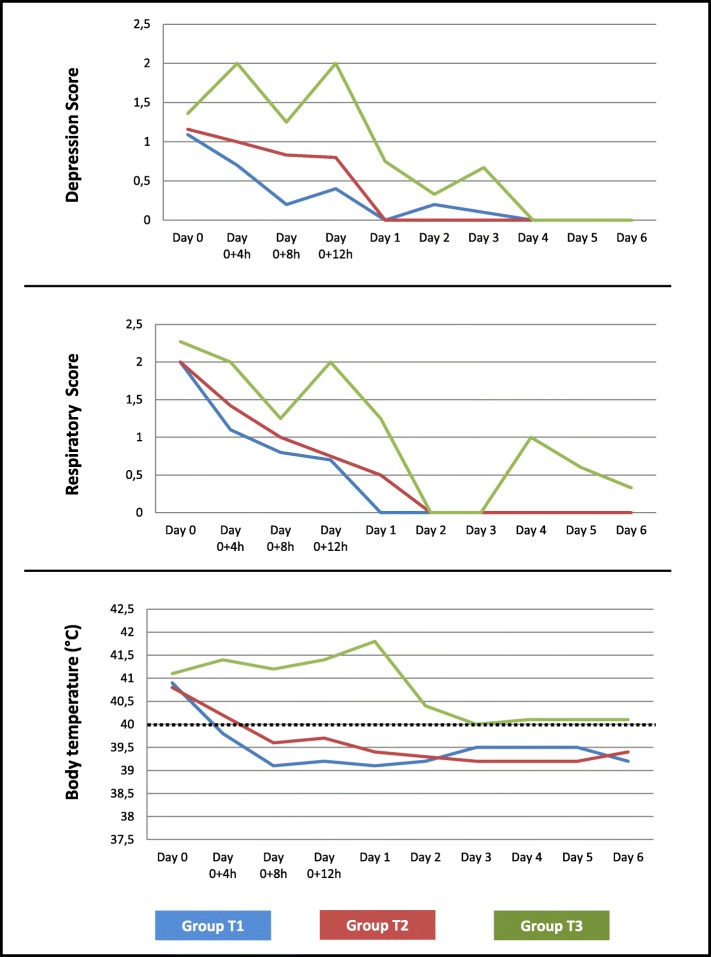
Clinical course of disease after treatment. Group T1: 8 mg/kg marbofloxacin, one-shot treatment, 11 pigs at Day 0, 10 pigs from Day 0 + 4 h onwards; Group T2: 2.5 mg/kg enrofloxacin, treatment on three consecutive days, 12 pigs; Group T3: 0.9% saline treatment, 11 animals at Day 0, 4 pigs from Day 0 + 4 h onwards, 3 pigs from Day 2 onwards; Day 0 = time of intention to first treatment, h = hours, Day 1 = 24 h after first treatment, Day 2–6 = 48–144 h after first treatment, dotted line = marking the threshold for physiological body temperature of 40.0 °C

Four hours after the first treatment, no animal in group T2, one pig in group T1 and seven in T3 had met the criteria for euthanasia. On day 2, one more pig in group T3 fulfilled the criteria for euthanasia and was removed, whereas no pig in group T1 or T2 showed any signs of clinical disease from 24 h after the first treatment onwards. On day 6 the respiratory score of one of the remaining pigs in group T3 was still 1 (Fig. 1). Overall, 8.3% (1 pig) belonging to group T1, 0.0% belonging to group T2 and 66.7% (8 pigs) belonging to group T3 were euthanized (Table 4).

A depression score of 2 was observed in none of the animals in T1, in two animals (16.7%) in group T2 and in all four remaining animals (100.0%) in group T3, four hours after treatment initiation. Eight hours after the first treatment, none of the animals in group T1 and T2 and one pig in group T3 showed a depression score > 1. The depression score was 0 for all animals in groups T1 and T2 at 24 h after first treatment and had returned to normal for the pigs in group T3 on day 4 (Fig. 1).

The rectal temperature returned to < 40.3 °C eight hours after treatment initiation in groups T1 and T2 and on day two in all remaining pigs of group T3. There was a small increase in temperature for less than 24 h on day three after the first treatment in group T1 and on day five after the first treatment in group T2 (Fig. 1). Regarding the course of disease, the differences were statistically significant between groups T1 and T3 as well as between groups T2 and T3 (*p* < 0.0001).

Of the ITT population, 10 pigs (90.9%) belonging to group T1, 10 pigs (83.3%) belonging to group T2 and 1 pig (9.1%) belonging to group T3 were considered cured on day 6 (Table 3). The difference was statistically significant between T1 and T3 and between T2 and T3 (*p* < 0.001).

The average daily weight gain (kg per day) also differed significantly (*p* < 0.05) between the groups T1 and T3 and between the groups T2 and T3 (Table 4).

### Lung lesion score (LLS)

The LLS was the primary efficacy criterion. As two pigs were treated despite not fulfilling all inclusion criteria only 32 pigs were included in this analysis (see Clinical data; Table 4). After log transformation of the data, a statistically significant superiority of T1 and T2 compared to T3 was proven (*p* < 0.0001; Table 4).

### Bacteriological examination

For the assessment of the bacteriological cure, the whole ITT population of 34 animals was included (Table 4). In group T1 *A. pleuropneumoniae* was re-isolated from the lungs from none (0.0%) of the pigs, in group T2 from one pig (8.3%) and from all pigs (100.0%) in group T3. Using the Fisher’s exact test, a statistically significant difference was confirmed between groups T1 and T3 and also between groups T2 and T3 (*p* < 0.001). There was no statistically significant difference between groups T1 and T2.

### Safety criteria

One animal of group T3 was found dead 24 h after treatment initiation. As this was the group treated with 0.9% NaCl-solution, the death was considered to be due to disease progression and not as a consequence of treatment.

No injection site reactions were observed in any of the treatment groups.

## Discussion

The results of this study show that treatment with marbofloxacin and enrofloxacin was significantly superior to the mock-treatment of 0.9% saline solution. Enrofloxacin was chosen as reference product for the positive controls because like marbofloxacin, it belongs to the second generation fluoroquinolones and has similar pharmacokinetic properties. Additionally, the selected product Baytril®- Das Original 50 mg/ml (Bayer Vital GmnH, Germany) is already registered for the treatment of *A. pleuropneumoniae* infection in swine. The significant reduction of the need to euthanize pigs due to the severity of symptoms confirms the importance of antibiotic treatment to treat and save infected pigs after the onset of clinical signs for animal welfare reasons. Both antibiotic treatment regimens (T1 and T2) induced a faster return to a normal rectal temperature as well as a faster recovery of the general condition and respiratory parameters. The observed minor fluctuation in body temperature observed in animals in groups T1 and T2 after treatment could probably be linked to changes in activity behavior of the pigs [38] but an hypothesis of relapse, even though less probable because of the absence of other clinical signs, could not be totally excluded. If the treatment was insufficient for example due to a short treatment period a relapse of infection and inflammation might occur accompanied by a repeated increase in body temperature. As the study was terminated on day seven post inoculation no definitive statement can be made if the increase would have led to a relapse of clinical disease or if it was only a short-term fluctuation.

Although there was a somewhat faster improvement of general condition, respiratory parameters and rectal temperature, there were no statistically significant differences between the marbofloxacin and enrofloxacin-treated groups. This also applies to the clinical cure, the lung lesion score and the bacteriological examination. Lung lesions were less prominent in the marbofloxacin-treated pigs. Isolation of *A. pleuropneumoniae* from the lung tissue was possible in a greater number of animals from the enrofloxacin-treated than from the marbofloxacin-treated animals although the difference was only one pig and is therefore not significant.

Comparing the results of this study with previous studies that also assessed the influence of fluoroquinolones on *A. pleuropneumoniae* infection, these results are comparable with the results from Grandemange et al. [39] who also tested the efficacy and safety of marbofloxacin and enrofloxacin for the treatment of porcine pleuropneumonia. The outcome of the Grandemange study also concluded that both antibiotics were equally effective. However, comparison of efficacy results between different studies should be made with care. It should be taken into account that small setup-related differences e.g. in applied infection dose, environmental conditions, used serotype or dosage may significantly influence the development of disease [4–6] and therefore the study results. Most published studies evaluating the efficacy of marbofloxacin on *A. pleuropneumoniae* infection are conducted either as field [33, 40] or in-vitro studies [41–44]. An advantage of experimental infections under standardized conditions is that confounding environmental factors, that are often discussed as reasons for failure or reduced efficacy in field studies, can be eliminated. Studies that evaluate the efficacy of marbofloxacin and enrofloxacin on porcine pleuropneumonia under experimental conditions [45–47] show cure rates of 80–100%. For the one-shot high dose marbofloxacin treatment of porcine pleuropneumonia an experimental counterpart eliminating the environmental factors has not been conducted before. Other studies evaluating the efficacy of marbofloxacin to treat porcine pleuropneumonia investigated the efficacy of dosages between 1.5 mg/kg and 5 mg/kg bodyweight, administered on four consecutive days [47]. Nevertheless the results are in accordance with the results of this study regardless of the fact that fluoroquinolones develop their main bactericidal activity in a concentration-dependent manner [31, 32].

Another fact is, that especially in field studies, where the exact time of infection of the individual animal cannot be determined; the efficacy of antibiotic treatment can be reduced due to the biofilm formation within the porcine lungs if treatment is initiated too late. It has been demonstrated that *A. pleuropneumoniae* takes active part in the biofilm formation [48]. Biofilms are a biopolymer matrix attached to the biotic surfaces produced by the local microflora. Bacteria, such as *A. pleuropneumoniae*, are able use such biofilms to shield themselves from the immune system or antimicrobial treatment due to a developing gradient of diffusion [49]. Biofilm formation starts a few hours after infection and can seriously affect the efficacy of administered antibiotics. Due to the acute infection in this study, the fact that the pigs were tested negative for *A. pleuropneumoniae* prior to inoculation and the early onset of treatment, it is unlikely that a protective biofilm matrix influenced the efficacy of either of the antibiotics.

In this study *A. pleuropneumoniae* could not be re-isolated from lung tissue of any of the marbofloxacin-treated animals, indicating that the bacterial cure of the lung tissue was 100%. Other related studies have already shown that enrofloxacin is also capable of eliminating *A. pleuropneumoniae* from lung tissue after controlled experimental infection [45, 46]. A main difference between these presented results and previous studies concluding that an antibiotic treatment (e.g. tulathromycin, tilmicosin) is unable to completely eliminate *A. pleuropneumoniae* from colonized pigs is that other studies used PCR protocols for the pathogen detection and also tested the tonsils of the infected pigs after treatment [18, 50]. Tonsils and evolved lung sequesters are the main locations described where *A. pleuropneumoniae* can survive despite a systemic antibiosis of the host [8, 51]. The PCR technique may also detect dead DNA, while culture depends on viability of the bacteria investigated [52]. Therefore conclusions regarding superiority or inferiority of fluoroquinolones for the elimination of *A. pleuropneumoniae* from infected pigs cannot be drawn based on the results of this study. Previous studies have demonstrated that marbofloxacin achieves a good penetration of tonsillar tissue and may reach sufficient concentration levels to remove *A. pleuropneumoniae* [53]. Nevertheless, no prediction of total clearance from animals can be made based upon the lack of isolation of bacteria from the lung tissue. Regarding the limitations of this study it should be mentioned that it only demonstrated the basic potential of this treatment procedure. A direct transfer of the results to field conditions cannot be made due to the diversity of the already mentioned environmental confounders and interactions with different microflora settings on different farms and within different animals.

Although the results of this study state a good efficacy and safety for the treatment of porcine pleuropneumonia with fluoroquinolones, the high importance of fluoroquinolones for use in human medicine should be stressed. It is therefore essential to limit the administration of fluoroquinolones to specific identified cases in order to maintain their efficacy and the low level of resistance which are equally important for their further use within the field of veterinary and human infectious diseases. Other measures such as improvement of husbandry practices and vaccination that have a major impact on the spreading of *A. pleuropneumoniae* infection [4] should be the method of choice when it comes to strategic containment of *A. pleuropneumoniae* leaving the use of fluoroquinolones for the treatment of clinical conditions that do not respond to other classes of antimicrobials.

## Conclusions

The one-shot 8 mg/kg marbofloxacin treatment was proven to be efficacious and safe for the treatment of porcine pleuropneumonia caused by an experimental aerosol inoculation, as confirmed by clinical, pathomorphological and bacteriological examination. A superiority of the marbofloxacin treatment compared to the 2.5 mg/kg enrofloxacin treatment administered on three consecutive days was not demonstrated. Nevertheless, the one-shot marbofloxacin treatment demonstrated the same efficacy as the three-shot enrofloxacin treatment while reducing the stress to the animals and the risk for administration errors due to the single administration. This study also demonstrated the importance of antibiotic treatment to reduce mortality during the acute phase of the disease compared to a mock-treated group.

## Abbreviations

*A. pleuropneumoniae*: *Actinobacillus pleuropneumoniae*
D6: Day six after first treatment
IM: Intramuscular
ITT: Intention-to-treat population
kg: kilogram
LLS: Lung lesion score
m: mean
mg: milligram
MIC: Minimal inhibitory concentration
p.inf.: Post infection
SD: Standard deviation
